# Dispersal strategies in the highly polygynous ant *Crematogaster (Orthocrema) pygmaea* Forel (Formicidae: Myrmicinae)

**DOI:** 10.1371/journal.pone.0178813

**Published:** 2017-06-07

**Authors:** Rachid Hamidi, Jean-Christophe de Biseau, Thomas Bourguignon, Glauco Bezerra Martins Segundo, Matheus Torres Marinho Bezerril Fontenelle, Yves Quinet

**Affiliations:** 1 Evolutionary Biology and Ecology, Université Libre de Bruxelles, Brussels, Belgium; 2 School of Life and Environmental Sciences, University of Sydney, Sydney, NSW, 2006, Australia; 3 Faculty of Forestry and Wood Sciences, Czech University of Life Sciences, Prague, Czech Republic; 4 Okinawa Institute of Science & Technology Graduate University, 1919–1 Tancha, Onna-son, Okinawa, 904–0495, Japan; 5 Laboratório de Entomologia, Instituto Superior de Ciências Biomédicas, Universidade Estadual do Ceará, Fortaleza, Brazil; University of Sussex, UNITED KINGDOM

## Abstract

In ants, dispersal strategies and morphology of female sexuals are generally linked to the mode of colony founding. In species using long-range dispersal tactics, queen/worker dimorphism is generally high and young queens are able to initiate new colonies by themselves, using their metabolic reserves. By contrast, in species using short-range dispersal strategies, queen/worker dimorphism is generally low and, due to their limited metabolic reserves, queens have lost the capacity to raise their brood alone and to found their colony independently. Moreover, polygyny is also often associated with short-range dispersal strategies, although the relationship between the number of queens and the dispersal strategy in ants is not clear-cut. Here, dispersal strategies were investigated in *C*. *pygmaea*, a highly polygynous and polydomous ant species from northeastern Brazil. Field observations and laboratory experiments show that this ant exhibits a suite of traits that are more commonly associated with long-range dispersal and independent colony foundation: functional wings in both males and females, high queen/worker dimorphism, strong weight loss in mature queens, nuptial flights and, in the lab, ability of young queens to found new colonies in haplometrotic conditions. On the other hand, this species shows a high degree of polygyny with a strong seasonal component, and, at least under laboratory conditions, mature queens seem able to develop propagules if they are accompanied by at least 10 workers. These features strongly suggest that (1) some of the gynes do not engage in a long-range dispersal but become new queens in their mother colony and (2) that budding events are possible in this species. We therefore speculate that *C*. *pygmaea* has a dual dispersal strategy probably related to environmental conditions: some gynes engage in long-range dispersal followed by independent colony foundation at the beginning of rainy season, while others mate in the parental colony and are re-adopted leading to high polygyny. During the rainy season, budding events can lead to colony extension and increased polydomy. Polydomy is commonly thought to improve resource discovery and exploitation through decentralized foraging behavior, a significant advantage during the rainy season when food ressources (mainly floral/extrafloral nectaries and hemipteran honeydew) are more abundant and when colony needs for food supplies are highest.

## Introduction

In ants, dispersal strategies in female sexuals are generally thought to be intimately associated with the mode of colony founding (independent colony foundation–ICF, and dependent colony foundation–DCF), and with distinct morphological traits in queens [1–5].

In ants with long-range dispersal tactics, males and females typically meet and mate during large mating flights that occur far away from the nests, and freshly mated queens settle new colonies without the help of nestmate workers (ICF) [1–3, 5, 6]. Except in primitive groups like poneromorph and myrmiciine ants, queens of species with a long-range dispersal strategy generally show strong dimorphism with workers (voluminous thoraces in queens compared to workers), especially in ants of higher ant subfamilies (*i*.*e*. Dolichoderinae, Formicinae, Myrmicinae) [1, 2, 4, 5, 7]. Young queens have enough lipid, protein (including flight muscles used as an amino acids source after muscles histolysis) and carbohydrate reserves to fly, dig a nest and rear the first clutch of generally nanitic workers [1, 2, 4–6, 8–11] losing up to half their weight during the process [8, 11, 12]. They also face a high predation rate, especially during swarming events and strong intraspecific competition, most of them dying before they can start reproducing [5, 6, 13, 14]. For instance, 80% of all incipient colonies of *Solenopsis invicta* perish before the first worker is able to forage [15]. To overcome these pressures, several young queens can cooperate after the nuptial flight and found a colony together (pleometrotic foundation and primary polygyny); this queens’ cooperation results in earlier and faster nest completion, and earlier emergence of the first workers produced in higher number, therefore increasing the competitive potential and the success of the newly established colony [6, 16]. However, primary polygyny generally ends in conflicts between queens, all but one being eliminated by workers (secondary monogyny) [16, 17].

In species with a short-range dispersal strategy, queen morphology and physiology generally differ greatly from that found in queens with long-range dispersal tactic: their wings are generally atrophied and non-functional or completely lost, and their metabolic reserves are too small to feed the first workers until they become able to forage; consequently, they have lost the capacity to raise their brood alone and to found their colony independently [2, 3, 5]. They therefore need the help of a group of nestmate workers to successfully found a new colony (DCF, with dispersal on foot) [1–3, 5, 18]. DCF is considered as a less risky strategy than ICF since it considerably decreases the risk of predation for queens and increases the competitive potential of incipient nests [1, 3, 5, 19]. It is nevertheless a potentially costly strategy since it necessitates the production of a worker force that will help founding queens [1, 3]. Size and composition of propagules (group formed by founding queen(s) and nestmate-worker helpers) are critical to the success of incipient colonies in DCF. For example, in the Argentine ant, larger propagules have a higher probability to survive and settle [20, 21]

Dispersal tactics are also thought to be associated with colony structure (number of queens), with single-queen (monogynous) and multi-queen (polygynous) colonies being associated with long-range dispersal (and hence ICF) and short-range (and hence DCF) strategy, respectively [22–25]. However, this traditional dichotomous division in monogynous/ICF-long-range dispersers and polygynous/DCF-short-range dispersers has since been challenged, as it seems that there is neither systematic relation nor causal link between DCF and polygyny, with DCF occurring in many monogynous species as well, across all subfamilies [1, 3]. Moreover, in some cases, like in polygynous species with polydomous systems, it may be difficult to disentangle what belongs to a colony founding strategy and to other ecological aspects of colony structure. In highly polydomous and polygynous species, short-range dispersal of female sexuals and the resulting polydomy could be, for example, a way to increase the number of nests and to therefore extend the territory of the colony (foraging strategy) [26, 27]

In this study, the dispersal patterns of a highly polygynous and polydomous neotropical species (*Crematogaster pygmaea* Forel, 1904) were investigated through analysis of morphological and behavioral traits generally associated to long-range dispersal/ICF (queen/worker dimorphism, weight loss in mature queens, ability to dig nests in absence of workers, success in founding colonies without the help of nestmate workers) or short-range dispersal (ability of propagules formed by mature queens and nestmate workers to develop). The occurrence of nuptial flights (long-range dispersal on the wing) was also investigated in field conditions.

*Crematogaster pygmaea* is a ground-nesting ant common in open, urban (or semi-urban) and sandy areas with sparse herbaceous vegetation of the state of Ceará (northeastern Brazil), the only place where this species has been found so far [28]. Its colonies are formed by tens of underground nests, each with several queens (mean of 4 queens/nest), and interconnected by surface trails used by workers to move from one nest to the other and to reach herbaceous plants where they collect nectar from floral or extrafloral nectaries, and/or honeydew from hemipteran colonies [28]. *Crematogaster pygmaea* colonies have a reduced number of nests and queens during the dry season, but they swiftly expand during the rainy season as the number of queens and nests increases [28, 29]. In two *C*. *pygmaea* colonies whose nest number was recorded every 4 to 5 months over one year (from March 2014 to April 2015), for example, nest number increased from 21 and 26 nests in October/November 2014 (end of dry season) to 143 and 64 nests in April 2015 (mid rainy season), respectively [29]. Such a population dynamic, characterized by seasonal polydomy and polygyny, is uncommon in ants [26, 30, 31].

Although the reproduction system of *C*. *pygmaea* is still unclear, it obviously differs from the general accepted pattern (but see [1, 3]), in which monogyny is associated with ICF and polygyny with DCF [22–24]]. Indeed, *C*. *pygmaea* paradoxically shares some characteristics with monogynous species. In particular, *C*. *pygmaea* is a multicolonial species (*i*.*e*. workers from different colonies show aggressiveness) [32] and queens show strong, although not yet quantified, dimorphism with workers [28, 33]. Furthermore, *C*. *pygmaea* queens have well developed wings and thorax, with enlarged mesonotum for attachment of wing muscles, big compound eyes and well developed ocelli [28, 33], all features that indicate good flight abilities and the probable existence of nuptial flights (and hence long-range dispersal) in that species. On the other hand, the highly polydomous structure of *C*. *pygmaea* colonies, the seasonal variation of colony polydomy [28], and the genetic structure of colonies (low genetic diversity of workers, despite high polygyny) [32] suggests that its reproductive strategy and/or foraging strategy also includes budding events (short-range dispersal).

*C*. *pygmaea* is therefore a good model to investigate the relationships between dispersal tactics, colony founding strategies, colony structure features (monogyny/polydomy, monodomy/polydomy), foraging and competition strategies, and their ecological correlates.

## Materials and methods

### Field sites

*C*. *pygmaea* is a common species in the littoral zone of the state of Ceará (Brazil) that belongs to the "caatinga" domain, an area of northeastern Brazil with a semiarid climate. Temperature is relatively stable all year-round, with an annual average of 26°C. Rainfall is low (less than 750 mm/year), mostly occurring during three consecutive months (generally from February to April) of the southern hemisphere summer (November to June). Male and gyne production (adults) mostly occurs at the end of the dry season and at the beginning of the rainy season respectively [28].

Nine large *C*. *pygmaea* polydomous colonies, all located in the littoral zone of the state of Ceará (northeastern Brazil), were used in this study, mainly to collect the workers and/or the female sexuals that were used in the experiments described hereafter. All colonies were found in anthropogenic (urban) and open areas with sparse herbaceous vegetation, the common habitat type of *C*. *pygmaea* [28].

Three colonies (Col-1, Col-2, Col-3) were located on the campus of the State University of Ceará (UECE) (3°47’ S– 38°33’ W), in Fortaleza. The distance between each pair of colonies ranged from ± 300 m to ± 800 m (Table 1). Three other colonies (Col-4, Col-5, Col-6) were also located in the municipality of Fortaleza, some 350 m (Col-4) or 3 km (Col-5, Col-6) away from the UECE campus (distance between Col-5 and Col-6: ± 800 m) (Table 1). The three last colonies (Col-7, Col-8, Col-9) were located in the municipalities of Caucaia (3°40’ S– 38°45’ W) (Col-7), Paraipaba (3°20’ S– 39°08’ W) (Col-8), São Gonçalo do Amarante (3°36’ S– 38°57’ W) (Col-9), and were distant some 26 km (Col-7), 80 km (Col-8) and 50 km (Col-9) from Fortaleza, respectively (the distance between each pair of colonies ranged from ± 25 km to ± 55 km) (Table 1).

**Table 1:** Distances (in km) between the field colonies used in the experiments.

Locality	Colony	Col-1	Col-2	Col-3	Col-4	Col-5	Col-6	Col-7	Col-8	Col-9
Fortaleza	Col-1	***								
Fortaleza	Col-2	0,8	***							
Fortaleza	Col-3	0,6	0,3	***						
Fortaleza	Col-4	0,35	0,6	0,35	***					
Fortaleza	Col-5	2,8	2,8	2,8	3	***				
Fortaleza	Col-6	3,4	3,4	3,4	3,6	0,8	***			
Caucaia	Col-7	26	26	26	26	26	26	***		
Paraipaba	Col-8	80	80	80	80	80	80	55	***	
São Gonçalo do Amarante	Col-9	50	50	50	50	50	50	25	35	***

No specific permissions were required to collect ants and/or make field observations in the study sites described above (all in public areas). The field studies did not involve endangered or protected species.

### Laboratory colonies

All the laboratory colonies used in the experiments described hereafter were kept in plastic containers (17 cm x 11 cm and 5 cm high, or 20 cm x 20 cm and 7 cm high, depending on the size of the colony) with inner sides coated with Fluon® to prevent ants from escaping. Glass test tubes (8 or 10 cm in length, 1 cm in diameter) provided with a water reservoir at the bottom of the tubes, and surrounded by a red plastic film, served as nesting sites for the colonies. The ants were fed *ad libitum* on sucrose solution and dead *Tenebrio molitor* larvae. The colonies were kept in an environmentally controlled room, with constant temperature of 30 ± 2°C and a 12:12 L:D photoperiod.

### Queen/Worker thorax volume ratio

In ants, queen/worker thorax volume ratios generally reflect the mode of colony founding [4, 7, 34]. This ratio was therefore assessed in *C*. *pygmaea*.

The thorax (i.e. alitrunk) length (mean of 3 length measures in side view for queens: superior, middle and inferior region; only one measure for workers), width (mean of 3 measures in upper view for queens: anterior, middle and posterior region; two measures for workers: wider and narrower region) and height (mean of 3 measures in side view for queens: anterior, middle and posterior region; only one measure for workers) was measured in 32 workers and 32 queens from five colonies (Col-1, Col-4, Col-5, Col-6, Col-7) (Table 2). The mean length, width and height were then used to calculate the mean thorax volume for queens and workers of each colony. Queen/worker ratio for each colony was then computed as the mean queen thorax volume divided by the mean worker thorax volume. Possible presence of different morphological types of queens (ex: microgynes, macrogynes) was also investigated.

**Table 2:** Queen/Worker thorax volume ratio (ratio Q/W) and mean thorax volume (TxV) (mm^3^) for queens and workers from five different *Crematogaster pygmaea* colonies.

Colony	Date of collection	TxV-Queens Mean ± SD	TxV-Workers Mean ± SD	Ratio Q/W
Col-1	IX/2010	2.418 ± 0.379^a^ (n = 10)	0.036 ± 0.009^a,c^ (n = 10)	67.3
Col-4	V and VI/2016	2.199 ± 0.200^a^ (n = 7)	0.039 ± 0.003^a,b^ (n = 7)	55.9
Col-5	V and VI/2016	2.450 ± 0.128^a^ (n = 5)	0.037 ± 0,004^a,b^ (n = 5)	66.39
Col-6	I/2017	2.305 ± 0,129^a^ (n = 5)	0.045 ± 0,005^b^ (n = 5)	51.29
Col-7	V and VI/2016	2.381 ± 0,189^a^ (n = 5)	0.041 ± 0,007^a,b^ (n = 5)	57.9

Means sharing the same letter in the queens or the workers group are not significantly different (level of significance α = 0.05) (Kruskal-Wallis test with post hoc Dunn test).

### Body weight in gynes and mature queens

Unmated winged queens of ant species with independent and claustral colony founding tactics generally store large amounts of fat and other substrates (carbohydrates, proteins) that are used in long-range dispersal flights and colony founding stage in claustral conditions [6, 8, 11]. Weight loss assessment in mated queens during founding stage can therefore be a good indication of dispersal abilities and of the mode of colony founding [8].

Mean fresh body weight of 67 gynes (here defined as unmated winged queens) that were collected in nests of four different colonies (Col-1, Col-5, Col-6, Col-8), at the beginning of rainy season of different years (2005, 2015, 2016) (Table 3), was compared to mean fresh body weight of 85 mature queens coming from four different colonies (Col-4, Col-5, Col-6, Col-7) (first six lines of Table 4).

**Table 3:** Mean weight of *Crematogaster pygmaea* gynes collected in different colonies and at different times (month and year).

Date of collection	Colony	Weighting date	N	Weight (mg) Mean ± SD
15/III/2005	Col-1	16/III/2005	10	10.9 ± 0.9^a^
15/III/2005	Col-8	16/III/2005	10	10.9 ± 0.9^a^
09/III/2015	Col-5	18/III/2015	4	10.8 ± 0.5^a^
21/II/2015	Col-6	20/III/2015	16	11.3 ± 0.7^a,b^
02/II/2016	Col-6	26/II/2016	18	10.7 ± 0.8^a^
04/I/2017	Col-6	06/I/2017	9	12.3 ± 0.5^b^

N: number of gynes. Means sharing the same letter are not significantly different (level of significance α = 0.05) (Kruskal-Wallis test with post hoc Dunn test).

**Table 4:** Mean weight of dealate queens obtained from lab mated *C*. *pygmaea* gynes or collected in the field, with age varying from one to several months since mating.

Date of collection	Colony	Fecundation date	Weighting date	N	Age	W-Q (gr) Mean ± SD	W-G (gr) Mean ± SD
09/III/2015 (1)	Col-5	15/III/2015	13/IV/2015	4	1	8.0 ± 0.5^a,b^	10.8 ± 0.5
21/II/2015 (1)	Col-6	15/III/2015	13/IV/2015	16	1	9.0 ± 0.8^b,c^	11.3 ± 0.7
27/V to 31/VI/2016 (2)	Col-7	?	22/02/2017	29	≥ 8	8.1 ± 1.2^a,b^	?
20 to 25/V/2016 (2)	Col-5	?	22/02/2017	12	≥ 9	8.5 ± 1.7^a,b^	?
21/VI to 10/VIII/2016 (2)	Col-4	?	22/02/2017	15	≥ 7	8.0 ± 0.9^a,b^	?
04/I/2017 (1)	Col-6	05/I/2017	06/02/2017	9	1	8.2 ± 1.0^a,b^	12.3 ± 0.5
02 to 29/III/2017	Col-5	?	31/III/2017	185	?	8.3 ± 1.0^a,b^	?
21/II to 15/III/2017	Col-4	?	06/02/2017	94	?	8.0 ± 0.8^a^	?

N: number of queens; Age: age of queens (in months) since mating; W-Q: weight of dealate mated queens; W-G: weight as gyne; (1) collected as winged queens (gynes) and mated in laboratory conditions; (2): collected as dealate and presumably fecundated queens. Means sharing the same letter are not significantly different (level of significance α = 0.05) (Kruskal-Wallis test with post hoc Dunn test).

All 85 mature queens had known age (from 1 to more than 7 months) since mating, either because they were obtained from gynes (n = 29) collected in the field (20 from Col-5 and Col-6 in 2015; 9 from Col-6 in 2017 –Table 4), mated in laboratory conditions and weighted one month later, or because they were from laboratory colonies where they were living since the time they had been collected (in 2016), in nests of Col-4 (n = 15), Col-5 (n = 12), or Col-7 (n = 29) (Table 4).

**Fig 1:**
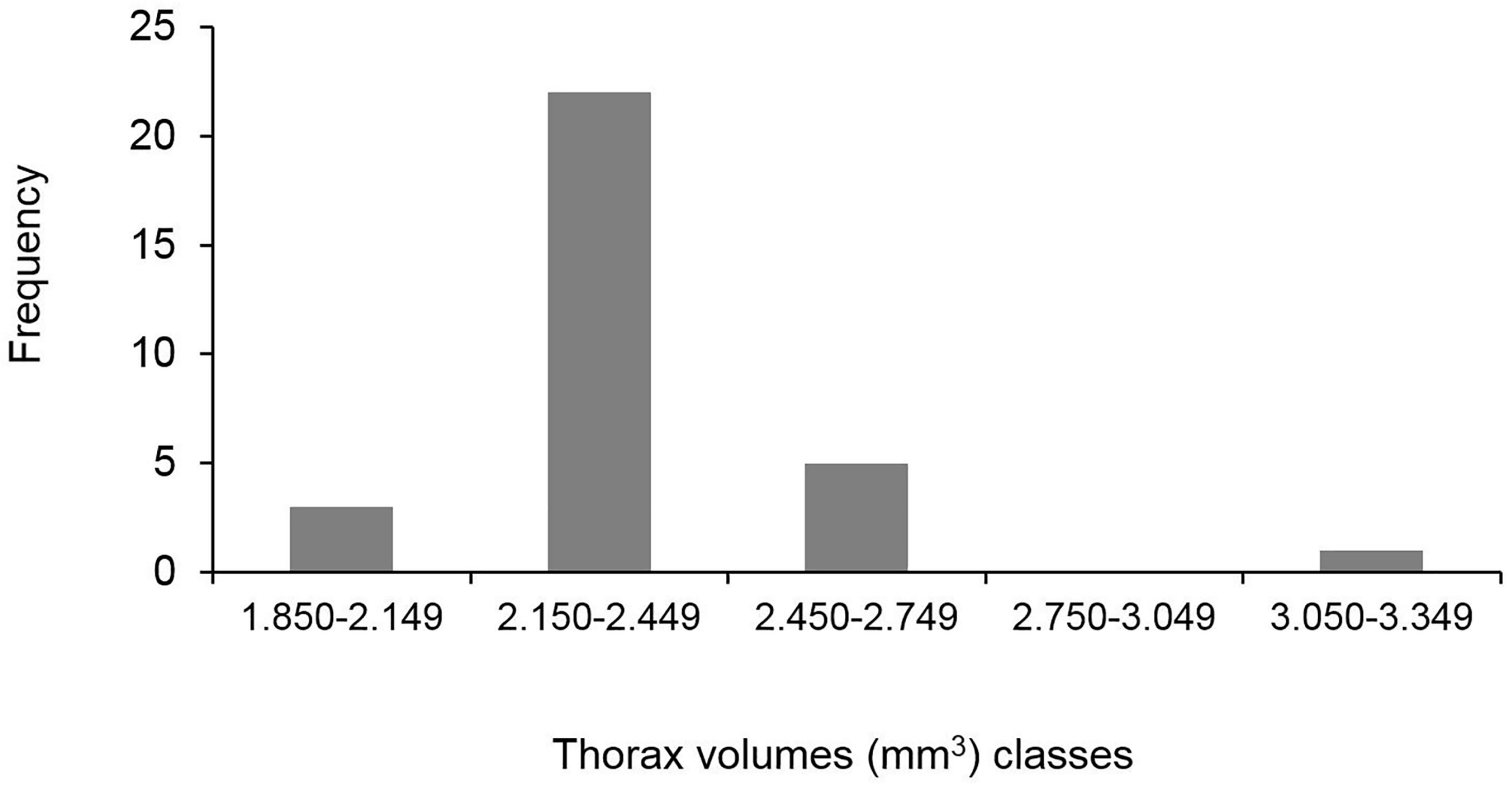
**Morphological differences between a gyne (unmated winged queen) (A,C) and a mature queen (B,D).** The gaster is yellowish in gynes while it is dark in mature queens.

Mature queens are here defined as queens that were at least one month old since mating. Minimum age for queens to reach maturity (one month) was arbitrarily established as the time necessary for young mated queens to rear the first clutch of adult workers in haplometrotic foundations, without helpers and food (independent and claustral conditions) (see results on development of haplometrotic foundations). Mature queens were also easily distinguished from freshly mated queens or gynes, by obvious differences in gaster morphology (especially gaster color) (Fig 1), as well as by their strong attractiveness to workers [35]. In order to document those morphological changes, images of gynes and mature queens gasters were generated using a Leica DMC2900 digital camera mounted on a Leica M205A stereomicroscope. Composite 3D images were assembled using Leica Application Suite (Version 4.5.0.) software package.

To obtain mated queens, gynes were individually placed, together with five males, in a plastic Petri dish (8.5 cm in diameter), with Fluon®-coated sides. Mating and dealation (wing shedding) behaviors generally occurred whithin a couple of hours. Soon after fertilization, each mated gyne was placed in a glass test-tube (10 cm in length, 1 cm in diameter) that was provided with a water reservoir at the bottom of the tube, surrounded by a red plastic film, and whose open end was closed by a cotton plug. The glass test-tubes were kept in a room with a constant temperature (30 ± 2°C) and a 12:12 L:D photoperiod, and were regularly checked to verify egg laying by queens, and, later, the presence of larvae, pupae and adult workers. After the first adult workers emerged, each young foundation was placed in a laboratory nest. The 29 queens mated in laboratory conditions (Table 4) were weighed two times: just before fecundation and one month after fecundation, when the first adult workers were produced.

Additionally, 94 and 185 queens were collected, in March 2017 (rainy season), in 12 nests of Col-4 and 22 nests of Col-5 (Table 4), respectively, and kept in laboratory nests, together with the other members of the excavated nests (queens, workers, brood). Although the age (since mating) of those queens was unknown, all had the morphology typically found in mature queens at the time they were collected (Fig 1). They were weighed on 31/III or 01/IV/2017, *i*.*e*. seven to 38 days after they were collected.

### Colony founding abilities in independent and claustral conditions

In ground-dwelling ants that have independent colony founding strategy, the ability of young mated queens to dig a nest and to rear brood alone, or in association with other queens, without the help of nestmate workers (independent colony founding conditions), is crucial for colony founding success [1, 6]. Those abilities were investigated in *C*. *pygmaea*.

The ability to dig a nest without the help of nestmate workers was assessed in three categories of *C*. *pygmaea* female sexuals: freshly mated (and dealate) young queens, mature queens (as indicated by their morphology at the time they were collected), and gynes. All female sexuals (96 gynes and 55 mature queens) were obtained from nests excavated in Col-1 between February and March 2005. Fifty-six gynes were used to obtain, in laboratory conditions, 56 young mated queens, using the same methodology described earlier.

The female sexuals were isolated, alone or in pairs, in round plastic boxes (12 cm in diameter and 15 cm high) whose bottom was covered with a 15-cm layer of sandy soil collected in the field, from places with *C*. *pygmaea* colonies. Twelve boxes and 22 boxes each contained one or two young mated queen(s) respectively; 15 boxes and 20 boxes each contained one or two mature queen(s) respectively; 20 boxes each contained two gynes. Twenty-four hours after isolation of the female sexuals, the number of nests dug in each box was recorded.

The ability of young queens to rear brood in haplometrotic or pleometrotic conditions was assessed in two series of experiments, with gynes of two different colonies (Col-6, Col-9).

In the first series, 49 gynes were collected in February 2005 (beginning of rainy season), in Col-9, and were mated in laboratory conditions, using the methodology described earlier. Nineteen and 30 of them were respectively placed alone (haplometrotic foundations; n = 19) or in pairs (pleiometrotic foundations; n = 15) in glass test-tubes (8 cm in length; 1 cm in diameter) provided with a water reservoir at the bottom of the tubes, surrounded by a red plastic film, and whose open end was closed with a cotton plug. Two months later, the number of workers found in each test-tube was recorded. Additionally, seven dealate young queens were collected in February 2005, at the end of a rainy day, wandering on the soil, some 300 meters from Col-1; they were probably recently fecundated gynes that landed after a nuptial flight. They were kept in glass nest-tubes (one queen per tube), under the same conditions as described above, and their ability to produce workers was observed two months later.

In the second series, eight gynes were collected on 04/I/2017 (beginning of rainy season) in Col-6, and were mated in laboratory conditions the next day. On 06/I/2017, each freshly mated queen was placed in a glass test-tube (10 cm in length; 1 cm in diameter) provided with a water reservoir at the bottom of the tubes, surrounded by a red plastic film, and whose open end was closed with a cotton plug. After the first adult workers emerged, each young foundation was placed in a laboratory nest regularly provided with food. The number of eggs, larvae, pupae and adult workers was recorded weekly from 13/I/2017 to 28/IV/2017 in each of the eight foundations.

### Worker size in incipient colonies

In ants with an independent and claustral colony founding strategy, the first workers to be produced in incipient colonies are typically undersized (nanitics) in relation to workers produced in older, mature, colonies [1, 6, 34, 36]. Presence of nanitic workers was therefore investigated in incipient colonies of *C*. *pygmaea*.

The size of four of the first workers produced in seven of the eight young haplometrotic foundations described above was assessed. In total, 28 workers were used to perform three types of morphometric measurements: head width (HW), *i*.*e*. the maximum width behind the eyes in full-face view; pronotal width (PW), *i*.*e*. the maximum width of pronotum in dorsal view; Weber’s length (WL), *i*.*e*. the maximum diagonal distance from base of the anterior slope of pronotum to metapleural lobe. The same morphometric measurements were performed with 100 workers coming from five mature laboratory colonies, each formed by queens, workers and brood collected in excavated nests of Col-3, Col-4, Col-5, Col-6, or Col-7. Twenty workers were collected in each laboratory colony to perform morphometric measurements.

### Propagules with mature queens

In *C*. *pygmaea*, queens are regularly observed on the trails of colonies, moving with small groups of workers, especially during the rainy season [29], when the number of queens and nests abruptly increases [28, 29]. Most of them are probably queens migrating from one nest to the other. However, such units formed by one or several queens and a small group of workers could also act as short-range dispersal agents (propagules), to found new colony units (nests), or even new colonies, as was shown with the propagules of some ant species, like *Linepithema humile* [20]. Development and survivorship of experimental propagules formed by mature queens and small groups of workers were therefore investigated.

Mature queens and workers collected in nests of Col-1 on 15/IV/2004 were used to form experimental propagules with differing numbers of queens and workers. Each propagule was kept in a plastic container (23 cm x 18 cm and 4 cm high), in laboratory conditions, with constant temperature of 30 ± 2°C and a 12:12 L:D photoperiod. Glass test-tubes (8 cm in length; 1 cm in diameter) provided with water reservoir at the bottom of the tubes and surrounded by a red plastic film, served as nesting sites. Ants were fed twice a week on sugar water (0.25 M sucrose solution), water and dry cat food.

The experimental propagules contained one, two, or four mature queens, and 0 (negative control), 10, 100, or 400 workers, following a fully crossed two-way design as described by [20]. Every treatment was replicated four times (in treatment with one and two mature queens) or five times (in treatment with four mature queens) (total of 52 experimental colonies). Queens, workers, and brood (eggs, larvae, pupae) present in each colony were counted on a monthly basis during four months. Development of each propagule was estimated using the sum of the number of eggs, larvae and pupae.

Fisher’s exact test and multiple logistic regression were used to assess how time, initial number of workers and initial number of queens affected queen mortality. The dependent variable in the multiple logistic regression was whether queen mortality occurred in a replicate. Correlation between brood production (summed numbers of eggs, larvae and pupae) and the number of workers per queen was tested using Spearman’s rank correlation. In addition, an exponential curve has been fitted to obtain number of workers per queen needed to reach the maximal brood production. The fit values were obtained using commercially software Origin 2016 purchased from Origin Lab Inc. (Northampton, Massachusetts).

### Nuptial flights

Based on previous indirect evidence of dispersal on the wing in *C*. *pygmaea* queens, occurrence of nuptial flights was systematically checked at the beginning of 2015 rainy season, in Col-6, a huge colony located in an area near the Zoological Garden of Fortaleza. Two groups of nests entrances (about 31 nest entrances in one group; about 19 nest entrances in the other group; the distance between the two groups was about 3 meters) interconnected by surface trails with heavy ant movements were surveyed every day from 16:00 to 19:00, during one week, from February 19, the first day with heavy rain (20 mm) in 2015 rainy season, until 27 February 2015.

## Results

### Queen/Worker thorax volume ratio

Mean queen and worker thorax volume varied from 2.20 (Col-4) to 2.45 (Col-5) mm^3^ and from 0.036 (Col-1) to 0.045 (Col-6) mm^3^ respectively (Table 2), giving an average thorax volume/worker thorax volume ratio varying from 51.3 (Col-6) to 67.3 (Col-1) (Table 2). Considering all queens (n = 32) and workers (n = 32) used to made measurements, mean queen and thorax volume was 2.35 ± 0.27 (SD) mm^3^ and 0.039 ± 0.007 (SD) mm^3^, respectively, giving an average queen thorax volume/worker thorax volume ratio of 60.23 (S1 File).

**Fig 2:**
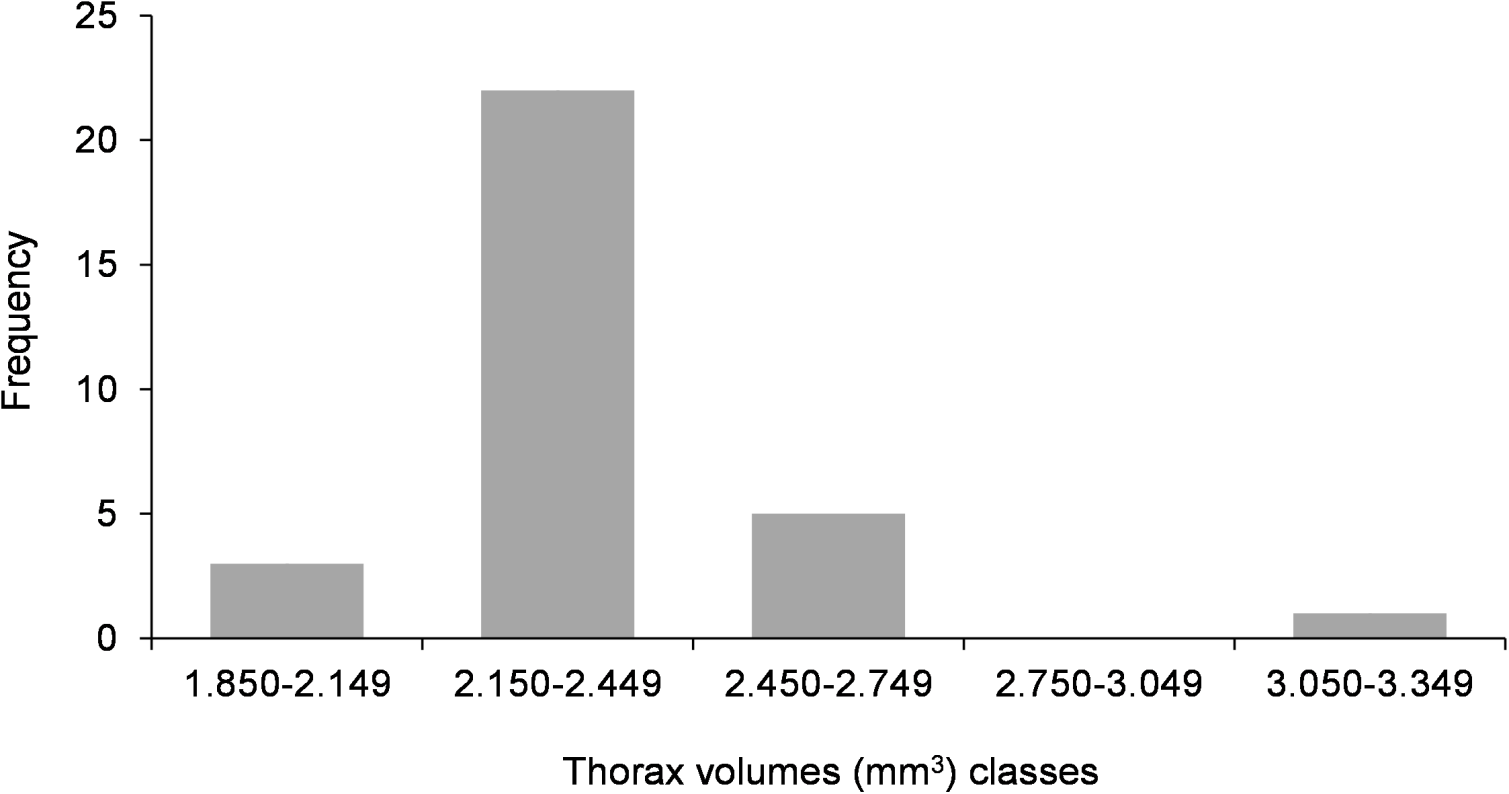
Frequency distribution of the queen thorax volume in *Crematogaster pygmaea* queens (n = 32) from five colonies.

**Fig 3:**
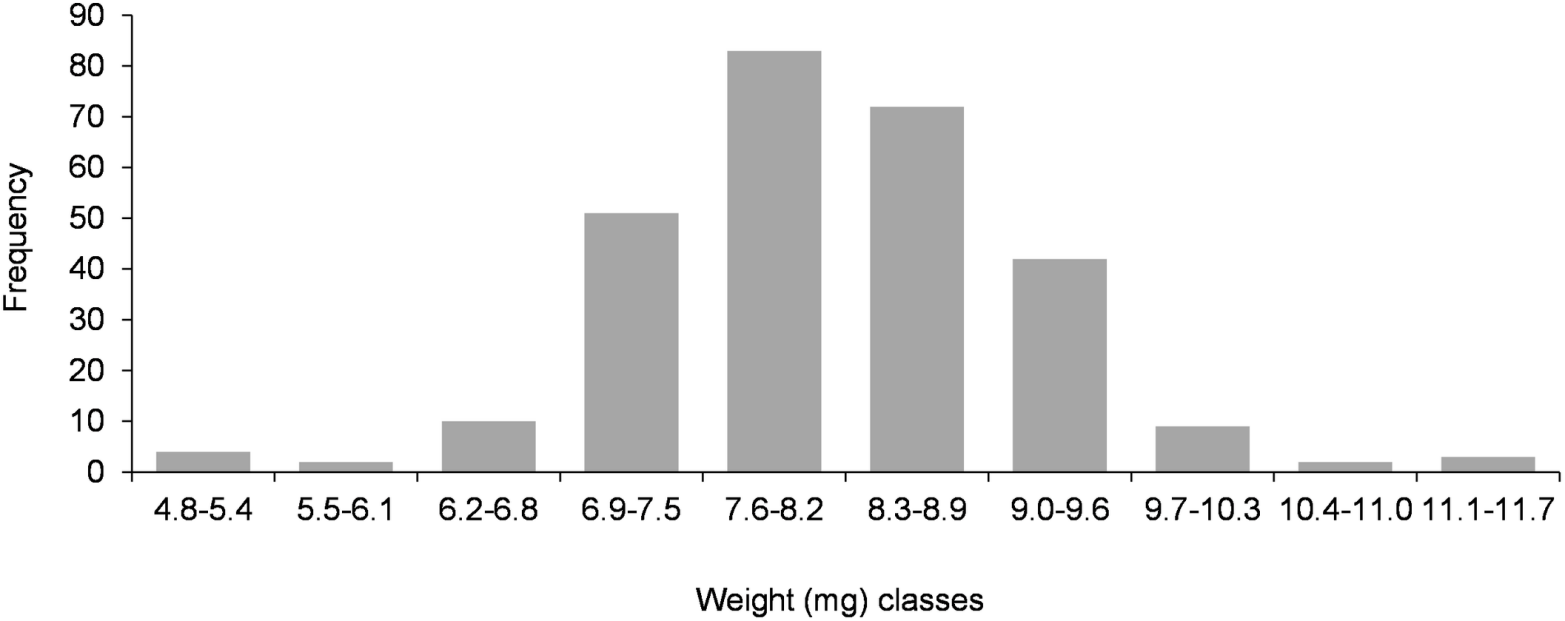
Frequency distribution of fresh body weight in *Crematogaster pygmaea* queens (n = 279) from two colonies (Col-4, Col-5).

No difference was found between the mean queen thorax volumes found in queens from the five colonies (p > 0.05—Kruskall-Wallis test) (Table 2), and the distribution of queen thorax volumes was found to be unimodal (Fig 2), *i*.*e*. with no signs of the presence of different morphological types of queens (ex: microgynnes, macrogynes), as also shown in Fig 3.

### Body weight in gynes and matures queens

In all mating experiments performed in laboratory conditions to obtain young mated queens, copulation was observed some 30 mins after the introduction of the gyne in the Petri dishes with males; all gynes shed their wings some 30 mins after copulation.

The mean fresh body weight of gynes varied from 10.7 mg (for gynes collected in February 2016, in Col-6) to 12.3 mg (for gynes collected in January 2017, in Col-6) (Table 3). No difference was found in the mean weight of gynes from the different colonies or time of collection, except for the gynes collected in January 2017 in Col-6, that were heavier than gynes collected in other colonies or times (except for gynes collected in Col-6, in February 2015) (Table 3).

In mature queens with known age since mating (from 1 to more than 9 months), the mean fresh body weight varied from 8 mg (for queens collected in Col-5 in 2015, or in Col-4, in 2016) to 9 mg (for queens collected in Col-6, in 2015) (Table 4). No difference was found in the mean weight of mature queens from the different colonies and/or age since mating (Table 4).

The mean fresh body weight for all gynes (n = 67) and mature queens (n = 85), independent of colony, collection time or age since mating, was 11.1 ± 0.9 (SD) mg and 8.3 ± 1.1 (SD) mg, respectively, giving a mean weight loss of 25.2% between gynes and mature queens (S2 File).

In the experiments where the weight of mature queens could be directly compared to the weight they had when they were unmated (gynes), weight loss varied from 21 to 33% (Table 4), with an average weight loss of 26%. In all experiments where queens were regularly followed from the moment of their fertilization, gaster morphology changed in the one month period after fertilization: the second half of gaster, that is yellowish in gynes, became dark (Fig 1).

The mean weight of the queens collected in March 2017 in Col-4 and Col-5, and of unknown age since mating, was 8.3 ± 1 (SD) mg and 8.0 ± 0,8 (SD) mg, respectively (Table 4), with no difference between the two colonies (p > 0.05—Mann-Whitney test). Considering all queens (n = 279), the mean weight was 8.2 ± 0.96 (SD) mg, a value similar to the mean weight obtained with mature queens of known age since mating (8.3 ± 1.1; n = 85) (p > 0.05—Mann-Whitney test). Furthermore, all had the gaster morphology found in mature queens. The distribution of queen weight in this group of 279 queens was found to be unimodal (Fig 3), like in thorax volume distribution (Fig 2). Again, no sign of the presence of different morphological types of queens was found.

### Colony founding abilities in independent conditions

Twenty-four hours after they were placed in plastic boxes with sand, all freshly mated (and dealate) queens placed alone in boxes (12 queens placed in 12 boxes) dug a nest. In all boxes where two freshly mated queens were placed (total of 44 queens placed, in pairs, in 22 boxes), at least one nest was dug: a common nest was dug by the two queens in half of the boxes (11/22); in the other boxes (11/22), each queen dug its own nest. Mature queens were not prone to dig a nest, since only two (13.3%) of the 15 mature queens placed alone in boxes dug a nest, against 100% for the young mated queens (p < 0.001—Fisher's exact test,). A similar result was obtained in boxes with two mature queens (total of 40 mature queens placed, in pairs, in 20 boxes), since only two boxes (10%) had at least one nest dug by queens. With gynes (total of 40 gynes placed, in pairs, in 20 boxes), the situation was intermediate since a nest was dug in nine boxes (45%). In gynes, the digging behavior seemed to be linked to the alate or dealate condition, since gynes which retained their wings did not dig nests, while gynes which shed their wings (although they were not mated) dug a nest.

Five of the 19 haplometrotic foundations (26%), and six of the 15 pleiometrotic foundations (two queens) (40%) formed with gynes collected in Col-9 and mated in laboratory conditions failed to produce any workers or were discarded, either because the queen (or one of the queens in foundations with two queens) died, or because phoretic mites invaded the queen(s) body. The remaining foundations headed by one queen (n = 14) or two queens (n = 9) produced between 24 and 44 nanitic workers (mean ± SD: 37.35 ± 4.98), and between 10 to 83 nanitic workers (mean ± SD = 47.00 ± 24.81) after two months, respectively (no difference in mean workers production was observed between to two types of foundation: p = 0.1681 –t-test) (S3 File). In pleometrotic foundations, no aggressiveness was observed between the queens, even after the emergence of the first adult workers who also didn’t show any sign of aggressiveness towards the queens. All young dealate queens collected soon after a probable nuptial flight (n = 7), and kept in haplometrotic isolation, dug a nest and produced workers.

**Fig 4:**
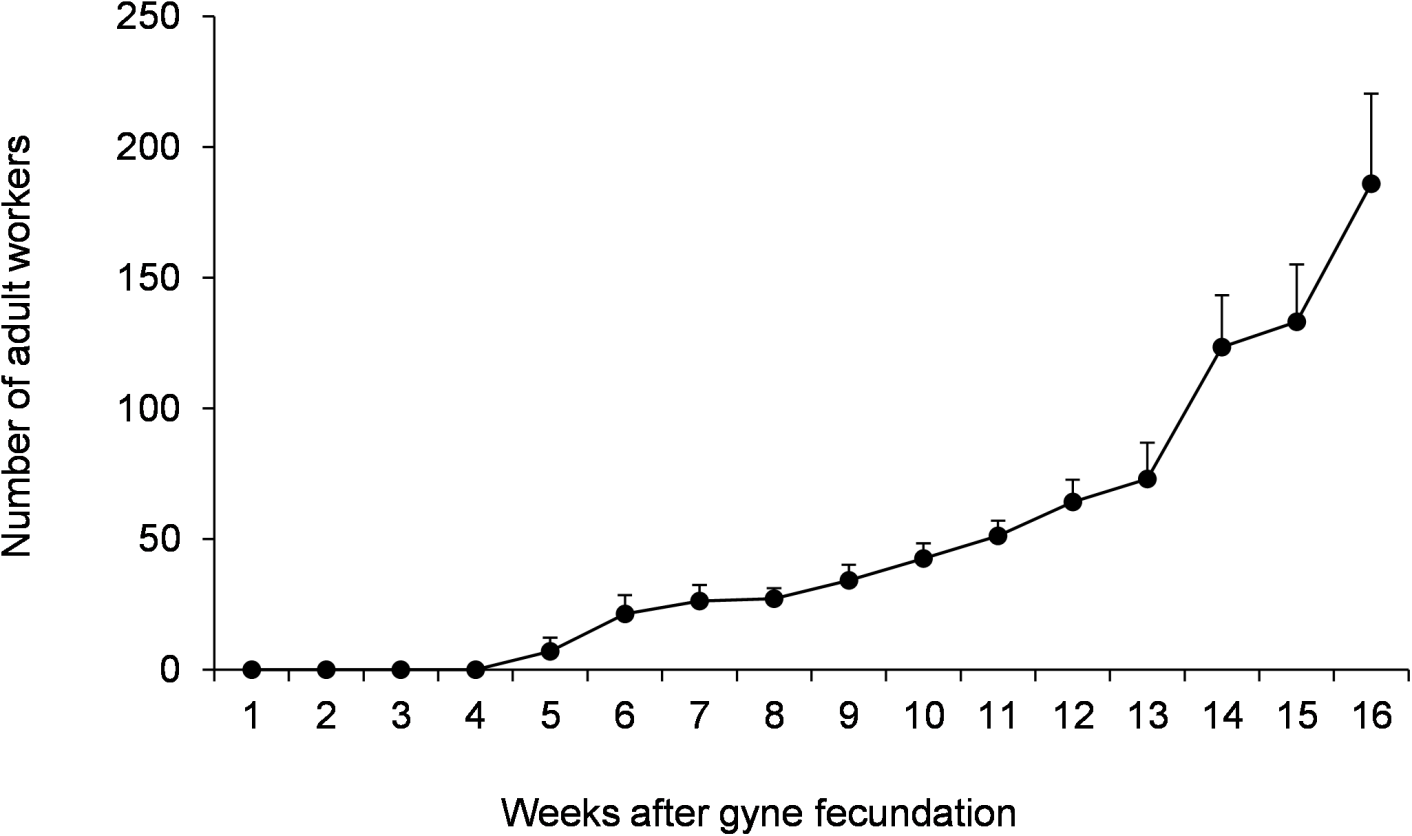
Mean number (± SD) of adult workers in *Crematogaster pygmaea* haplometrotic foundations (n = 8) 1 to 16 weeks after gynes fecundation.

All gynes collected in January 2017 in Col-6 (n = 8) and mated in laboratory conditions began to lay eggs soon after they were isolated in glass-test tubes (one young mated queen per tube). In all foundations, the first larvae, pupae and (nanitic) adult workers emerged two, four and five weeks after gynes fecundation, respectively (S4 File). Worker population regularly grew in all incipient colonies, with worker size population reaching a size of about 35 (mean ± SD: 34,25 ± 5,97; n = 8) and 190 individuals (mean ± SD: 186 ± 34,5; n = 8), 9 weeks (2 months) and 16 weeks (3.7 months) after gynes fecundation, respectively (Fig 4).

### Worker size in incipient colonies

All morphometric measurements performed with the first workers produced in seven incipient colonies (haplometrotic foundations) gave values (means ± SD) smaller (HW: 0.408 ± 0.007 mm; PW: 0.300 ± 0.007 mm; WL: 0.464 ± 0.009 mm—n = 28) than those obtained with workers from five mature colonies (HW: 0.491 ± 0,025 mm; PW: 0.345 ± 0.018 mm; WL: 0.566 ± 0.034 mm—n = 100) (p < 0.0001 for HW, PW and WL–Mann-Whitney U test). On average, the first workers to be produced in an incipient colony were therefore ± 20% smaller than those produced in mature colonies (S5 File).

### Propagules with mature queens

**Fig 5:**
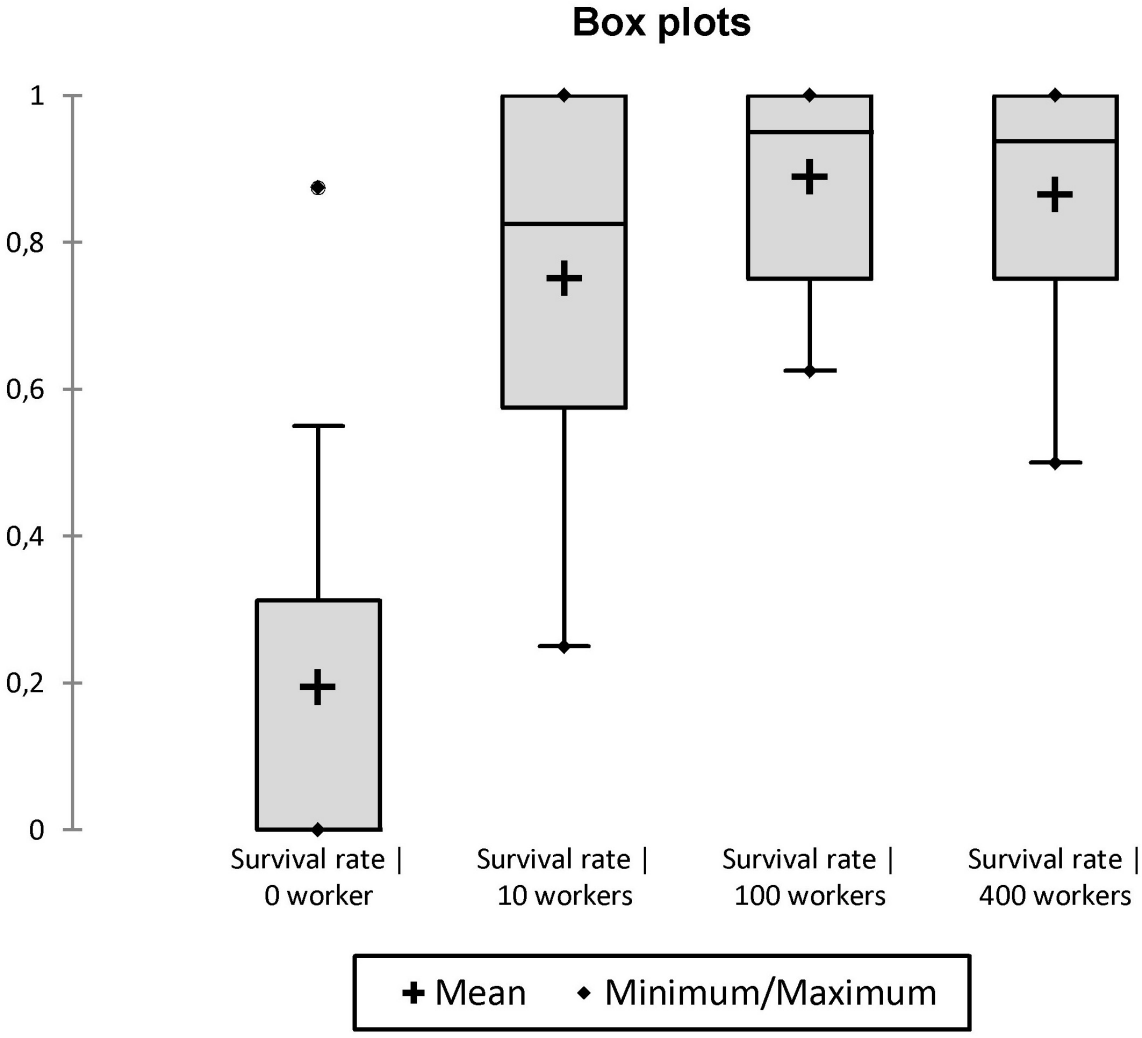
Box plot comparing the effect of initial number of workers on the survival rate of queens.

**Fig 6:**
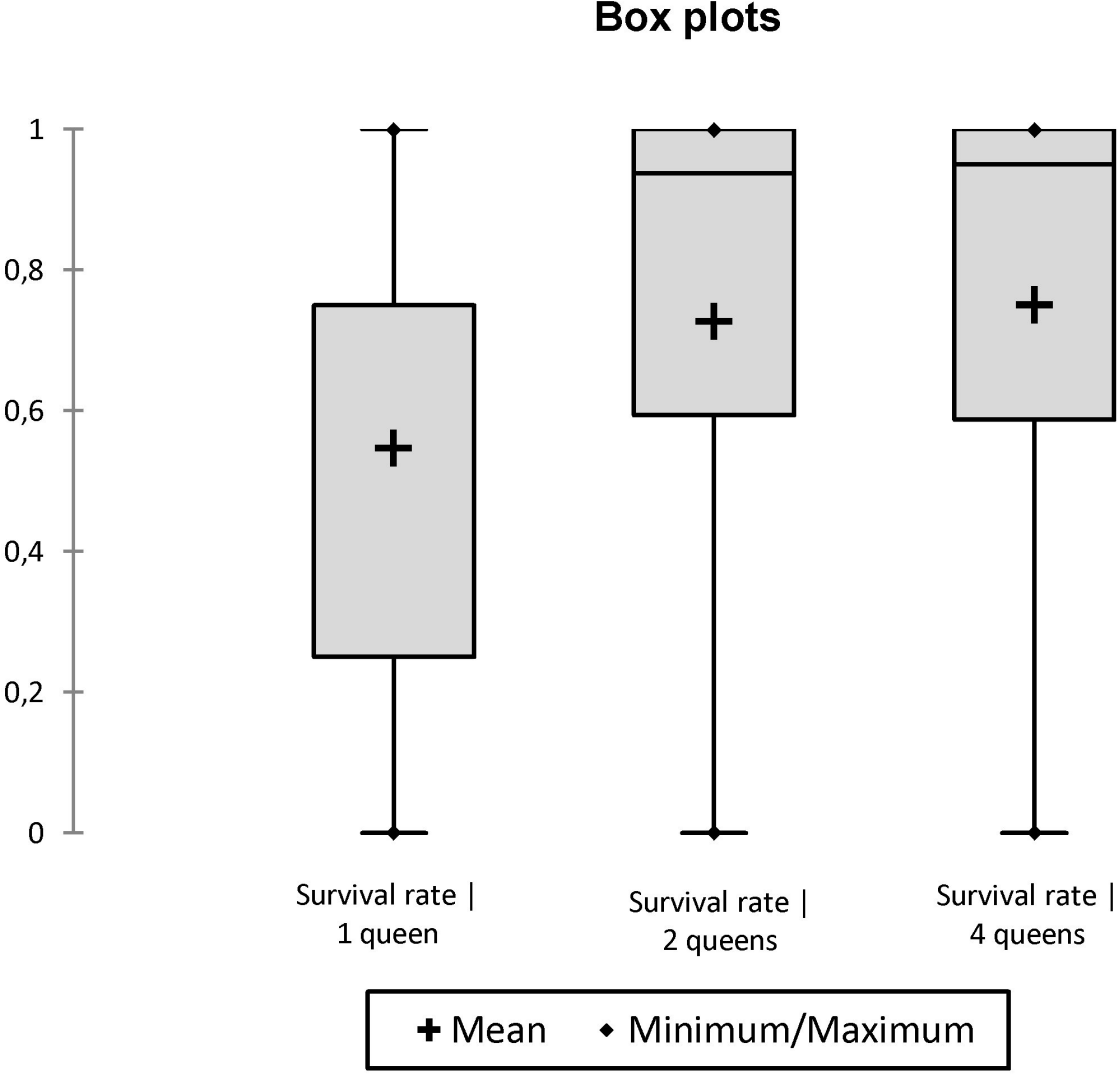
Box plot comparing the effect of initial number of queens on the survival rate of queens (including groups without workers).

**Fig 7:**
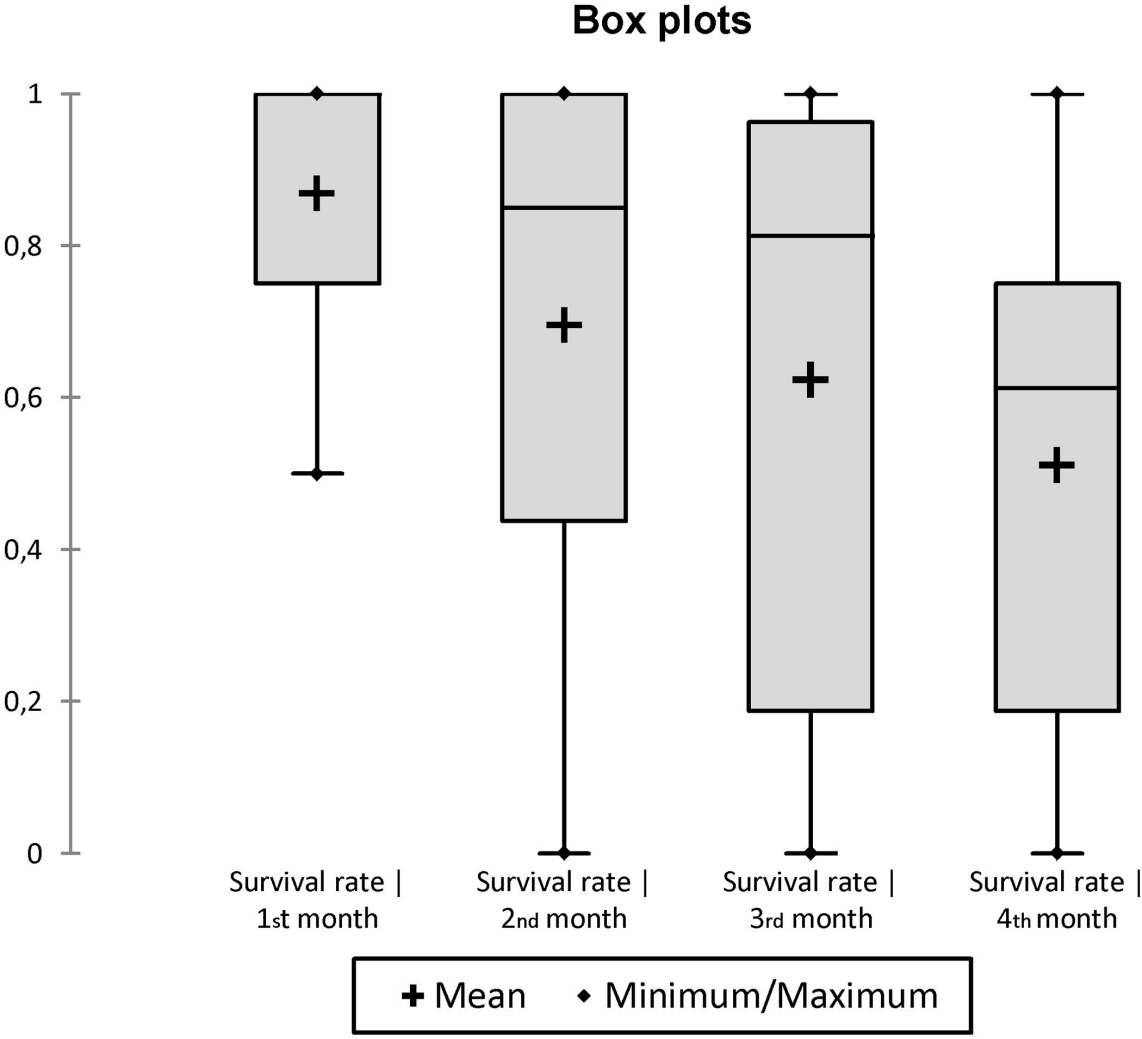
Box plot comparing the effect of time on the survival rate of queens (including groups without workers).

In propagules initiated without workers, the eggs laid by mature queens never hatched. After one and two months, only 62.5% (20/32) and 12.5% (4/32) of queens were still alive, respectively. After three months, no queens were alive. In propagules initiated with workers (10, 100 or 400 workers), the mortality of queens was significantly reduced: 2% (2/96) and 19% (19/96) of queens died after one and four months, respectively. Although the presence of workers influenced queen survival, their number did not. Indeed, about half of the variation in mean rate of queen survival is accounted for by the multiple regression model (R^2^ = 0.47, n = 36, AIC: -128.60, Whole-model F = 9.68, df = 3, p = 0.0001). In this model, queen survival was independent of worker number (r = 0.0001, t = 1.56, df = 1, p = 0.2201) but positively and significantly influenced by the number of queens (r = 0.0818, F = 14.82, df = 1, p = 0.0005) (Figs 5 and 6). Moreover, the time affected negatively and significantly queen survival (r = -0.0844, F = 14.82, dl = 1, p = 0.0011) (Fig 7).

**Fig 8:**
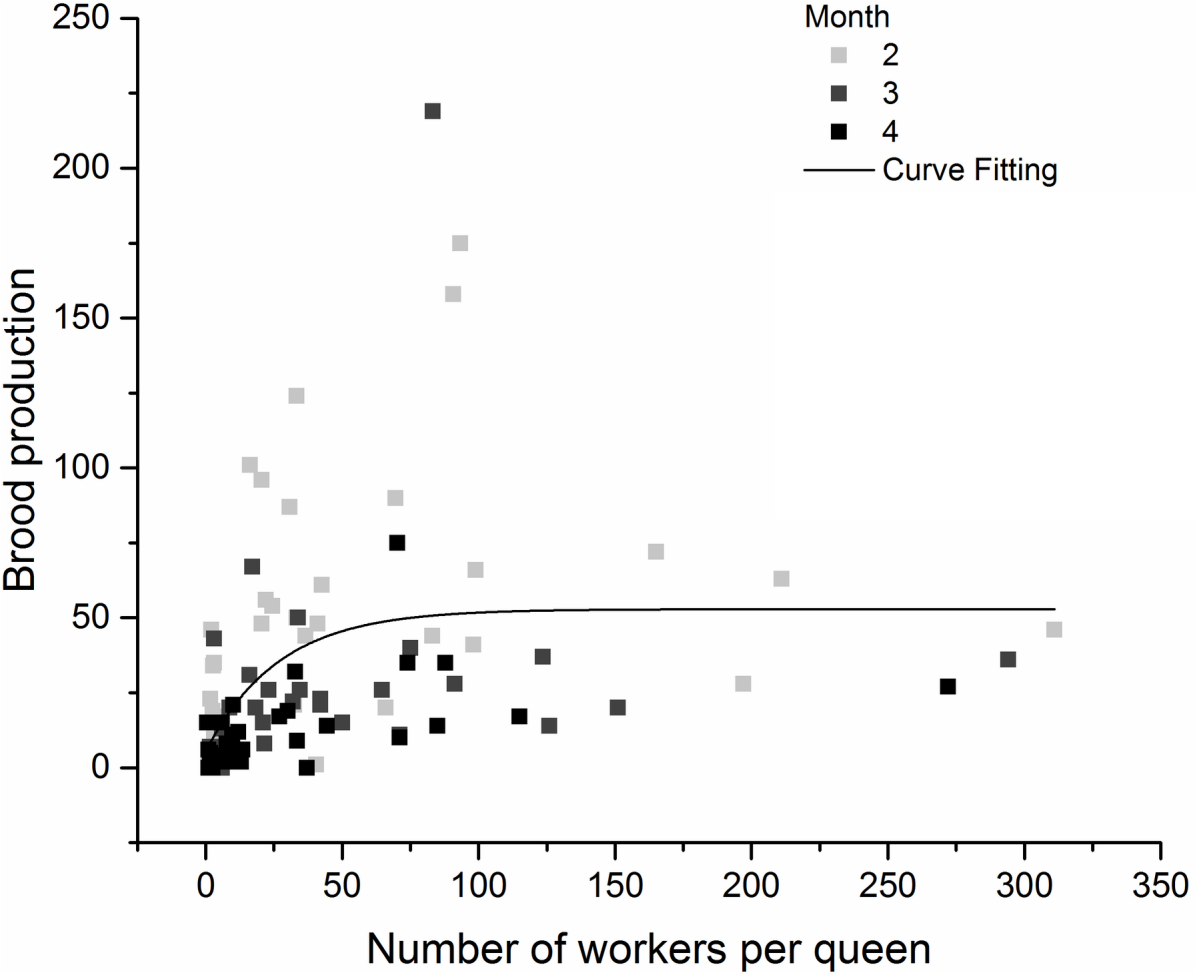
Effect of the number of workers per queen on the brood production. Model: ExpDec2; Equation: y = A1*exp(-x/t1) + A2*exp(-x/t2) + y0; y0 = 52.89 ± 12.00; A1 = -24.18; t1 = 26.80; A2 = -23.20; t2 = 26.78; Reduced Chi-Square = 1148.78; R-Square (COD) = 0.199; Adj. R-Square = 0.166.

A positive correlation was observed between the total brood (sum of the numbers of eggs, larvae and pupae) produced and the number of workers per queen, both in the second month (Spearman’s rank correlation coefficient, r = 0.4864, p = 0.0026), and after four months (Spearman’s rank correlation coefficient, r = 0.5938, p = 0.0004) (Fig 8). In addition, an asymptotic curve (obtained using the correlation between the number of workers (real number of workers present in the colony in the fourth month) per queen and the total brood produced during the fourth month) suggest that a minimum number of 50 workers per queen is necessary to allow a maximal brood production (R^2^ = 0.19; Offset (yo) = 52.89 ± 12.00). When a linear fit is tested, R^2^ is lower (R^2^ = 0.07) (Fig 8).

### Nuptial flights

Nuptial flights in ants are difficult to observe, since they generally occur during a short period of the year, with a species-specific diel rhythm generally associated with climatic parameters, like rain events, air humidity and/or wind speed [6, 37].

Our first observations were casual. On 16 February 2005, for example, at the end of the day, two days after the first heavy rain of 2005 rainy season, many gynes were observed climbing on the wall of a building, apparently attracted by a light source; some of them were copulating with a male. Dealate gynes were also observed wandering on the ground nearby, and one of them was seen trying to dig. The building was located some 300 meters from Col-1.

A more systematic observation was done on 22 February 2015, two days after the first day with heavy rain of 2015 rainy season. A large nuptial flight was observed at the end of the day, at the two surveyed groups of nest entrances in the *C*. *pygmaea* colony located near the Zoological Garden of Fortaleza. It happened after one night and one morning period with moderate to heavy rain. Nuptial flight began at ± 04:30 p.m., with winged queens emerging sporadically but regularly from six nest entrances (three in each surveyed groups of nest entrances). Most of them returned almost immediately inside the nests before soon emerging again, in an excited way. Those in and out movements of gynes were observed for about one hour, until ± 05:30 p.m., when plenty of gynes suddenly emerged from the nest entrances in a very excited way, running in an area about 15 cm in diameter around each nest entrance, together with many very excited workers. Gynes then began to climb up and down on herbaceous plants near the nest entrances, and at ± 05:45 p.m., a lot of them began to take flight. Many gynes also took flight directly from the ground, without climbing on plants. Flight was very powerful, with gynes flying fast and up, all in the same direction. Fifteen minutes later, the number of gynes taking flight began to strongly decrease. On 06:15 p.m., most gynes that had not taken flight had returned inside the nests. Some twenty minutes later, no more gynes were observed outside the nest and normal worker activity around the nest entrances was back. No males were observed during this nuptial flight. On the following days, some in and out movements of gynes could be observed again at some nest entrances (no males observed), at dusk and at the beginning of night, but no more nuptial flights occurred.

## Discussion

Our results show that *C*. *pygmaea* has morphological and behavioral traits that are found in many monogynous species of higher ant subfamilies (Dolichoderinae, Formicinae, Myrmicinae) with long-range dispersal tactic and an independent and claustral founding strategy.

Morphologically, *C*. *pygmaea* shows a high degree of dimorphism between queens and workers, with a queen-worker thorax volume ratio (± 60) in the range of, and even much higher than those found in well know monogynous and independent nest founders like *Lasius psammophilus* (31), *Lasius flavus* (25) and *Lasius niger* (24) [7]. It is similar to that found in *Crematogaster abstinens* (± 62 –Y Quinet pers. com.), a monogynous species (Y Quinet pers. com.) very close to *C*. *pygmaea* and occurring in sympatry with it in the Ceará state (northeastern Brazil) [28]. The observed weight reduction from young fertilized queens to mature queens (about 25%) also falls within the range of traits observed in independent and claustral colony founding species: in monogynous ants, the weight of queens after fertilization and founding phase may be reduced by 16 to 50% [8, 9, 17, 38–41]. This voluminous thorax and the significant weight loss in mature *C*. *pygmae*a queens are therefore in line with the ability of ant queens to sustain powerful flight (long-range dispersal on the wing with enlarged wing muscles and carbohydrate reserves) and to rear their first brood with metabolic reserves (fat stores, storage proteins, amino acids from histolysed wing muscles) [2, 4, 7, 8].

The ability of young mated queens, at least in laboratory conditions, to dig a nest and to produce a first batch of nanitic workers in haplometrotic conditions, without any external food supplies, and the loss of this ability in mature queens, is another strong indication that *C*. *pygmaea* gynes have, like gynes of many monogynous species, enough metabolic reserve to found colonies in an independent and claustral way [1, 2, 6].

Production of nanitic workers from the first brood is considered as an adaptive trait in ants with independent and claustral founding strategy [34, 36, 42]. Small size of *C*. *pygmaea* workers in adult colonies is also a good indication of founding mode, since very small workers in adult colonies (“dwarf workers” *sensu* [34]), relative to queens, is also a morphological trait considered to be adaptive in ants with an independent and claustral founding mode [34].

Thorax architecture in queens is another morphological trait that strongly supports independent and claustral founding strategy in *C*. *pygmaea*. As shown by [4], two types of queen thorax architecture have originally evolved in ants: one associated with a non-claustral independent founding mode (probably the ancestral condition), the other associated with claustral independent founding mode, this last one having further evolved in ants with a dependent founding mode. Thorax architecture of *C*. *pygmaea* queens undoubtedly belongs to the “claustral independent founding mode” category, with second segment (mesotonum) that is greatly enlarged in order to attach huge wings muscles that are used in flight and the founding phase, and first segment (pronotum) greatly reduced [33].

Last but not least, the occurrence of large-scale nuptial flights (at least in female sexuals) and casual observations of gynes copulating with males in places distant from established colonies are also indications for the existence of a long-range dispersal tactic in *C*. *pygmaea* [1, 6].

Those “monogynous” traits are, nonetheless, very surprising in view of the high degree of polygyny found in *C*. *pygmaea* colonies [28, 32], since polygyny generally results from an opposite reproductive strategy, namely the adoption/re-adoption, in an established colony, of newly mated queens coming from other colonies after long-range dispersal with mating flights (adoption), or of young queens that mated close to or, even, inside the parental nest (re-adoption) [6, 43, 44]. As proposed for other highly polygynous ant species like *Anoplepis gracilipes*, *Plagiolepis pygmaea*, or *Pseudomyrmex peperi* [45–49], or polygynous species of the *Formica* group [24, 25, 50], polygyny in *C*. *pygmaea* probably results from the re-adoption, in the parental colony, of young females that mated within it (inside the nests or close to them). Such a hypothesis is supported by the genetic data obtained in a previous study: using the analysis of polymorphic microsatellites, this study showed that the workers of *C*. *pygmaea* colonies are highly related (mean index of relatedness (r) between workers of a colony: 0.58) despite high polygyny, thereby indicating strong relatedness among queens within the same colony [32].

However, mating in the parental nest/colony, or close to it, does not mean staying in that nest/colony. Indeed, in many ant species whose queens mate within the parental colony, the newly mated queen(s) leave(s) the parental nest/colony with a group of nestmate workers and found a new autonomous colony, at some distance of the parental colony (short-range dispersal and DCF) [3, 51]. Alternatively, the newly mated queens can leave the parental colony with a group of nestmate workers and establish a new nest that remains in contact with the parental nest, therefore becoming a new unit (nest) of a polydomous and polygynous colony [1, 26].

Previous data and present results strongly suggest that part of the gynes produced each year at the beginning of the rainy season in *C*. *pygmaea* colonies do not engage in mating flights (long-range dispersal), but rather mate in the parental colonies, and engage in budding events, consequently increasing polygyny and polydomy of colonies. First, *C*. *pygmaea* colonies undergo a strong and swift expansion (increase in number of nests and of area occupied by colonies) at the beginning of the rainy season, at the same time that many new gynes are produced [28, 29]. Second, queens (or gynes) were frequently observed moving between nests of colonies during the rainy season, although the unpredictability in time of such events makes it difficult to investigate the regularity, frequency and details of such behaviors [1, 51]. Finally, the ability of propagules formed by at least one mature queen and 10 workers (the optimal number being between 50 and 100 workers) to develop, also indicate the possibility of budding events.

It seems therefore that *C*. *pygmaea* has a dual dispersal strategy: a long-range dispersal strategy through mating flights and a short-range dispersal strategy through budding events that follow fecundation of gynes in the parental colony. A similar dual dispersal strategy has been found in other ants, one of the best-known example being that of wood ants of the *Formica rufa* group [24, 25, 50]. According to Rosengren et al. [24], risky dispersal and low probability of independent founding (e.g. habitat patchiness) are key factors for selection against long-range dispersing phenotypes and, consequently, for polygyny through re-adoption of young queens and budding. In *C*. *pygmaea*, this dual dispersal strategy would not be based on a queen polymorphism (ex: micro and macrogynes), like in other ant species [2, 52], since such polymorphism was not detected.

In *C*. *pygmaea*, we speculate that while the long-range dispersal behavior would be part of a colony foundation strategy, probably with independent colony foundation (ICF) in haplometrotic or pleometrotic conditions, the short-range dispersal behavior would be part of a foraging strategy (seasonal polydomous system).

*Crematogaster pygmaea* lives in an anthropogenic, disturbed and, in many cases, urban or peri-urban habitat; this habitat consists of small to medium sized habitat patches (sandy areas with sparse herbaceous vegetation), isolated by much larger areas with trees and/or shrub vegetation, and/or dense herbaceous vegetation [28]. Furthermore, the whole region where *C*. *pygmaea* is found (northeastern Brazil) has a semiarid climate with rainfall concentrated in three consecutive months of the year (mainly from January/February to March/April) and dry season during the rest of the year [53]. This harsh climatic environment is prone to add more instability to the anthropogenic habitat patches where *C*. *pygmaea* colonies are generally found. The long-range dispersal strategy could therefore represent a way to escape from habitat instability (ex: deteriorating habitat) or saturation, allowing dispersers to enter new suitable patches to found new colonies. The lack of aggressiveness between young queens in pleiometrotic foundations, even after the emergence of the first adult workers, suggests that new *C*. *pygmaea* colonies could be founded by several queens and that, once established, the young colonies could retain all the founding queens (primary polygyny).

On the other side of dispersal scale, the short-range dispersal strategy would be a way, not to found a new colony through DCF, as shown in many species with short-range dispersal [1, 3], but to expand the nest network of the colony (increased polydomy) when food sources (herbaceous plants with floral/extrafloral nectaries and/or hemipteran colonies) are more abundant (rainy season) and when need for food sources is high (more brood to feed). One of the most cited adaptive benefits of polydomy is improved foraging organization through resource discovery and exploitation benefit (energetic savings through decentralized foraging behavior) [26, 27]. New colonies could, nevertheless, be created during the expansion phase of colonies (rainy season), or even during the retraction phase of colonies (during the dry season), should a group of nests became accidentally separated from the rest of the colony (opportunistic DCF *sensu* Cronin et al. [3]).

We speculate that the dual dispersal strategy of *C*. *pygmaea* arose from a monogynous and monodomous ancestor, through an evolutionary modification that resulted in high tolerance between related queens and secondary polygyny through re-adoption of young queens after intracolonial mating. The resulting polygynous system would have made easier the emergence of a polydomous colonial structure able to track seasonal variations of food sources abundance and distribution, in patchy and man-shaped habitat with strong seasonal variation of food resources availability (semiarid environment). *C*. *pygmae*a would have retained from the monogynous and monodomous ancestor the long-range dispersal strategy, allowing colonization of new patches and/or escaping from patches instability. Tolerance between related queens would have also allowed permanent association of young founding queens (primary polygyny), allowing a quick development of new colonies. Comparison of *C*. *pygmaea* biology with that of highly related species could help to understand the evolution of polygyny and polydomy in *C*. *pygmaea*. *Crematogaster abstinens*, a very close species that occurs sympatrically with *C*. *pygmaea* in the littoral region of Ceará state [28] could be a good candidate since field investigations showed that it is a monogynous and monodomous species (Y. Quinet pers. com.).

## Supporting information

S1 File Queen/Worker thorax volume ratio. (XLSX)

S2 File Weight of gynes and mature queens. (XLSX)

S3 File Independent foundation and propagules development in *C*. *pygmaea*. (XLS)

S4 File Foundation development in *C*. *pygmaea*. (XLSX)

S5 File Morphometric data for nanitic workers in *C*. *pygmaea*. (XLSX)
