# Correction: Dispersal strategies in the highly polygynous ant *Crematogaster (Orthocrema) pygmaea* Forel (Formicidae: Myrmicinae)

**DOI:** 10.1371/journal.pone.0185878

**Published:** 2017-09-28

**Authors:** The PLOS ONE Staff

Fig 1 is incorrect. The authors have provided a corrected version here. The publisher apologizes for the error.

**Fig 1:**
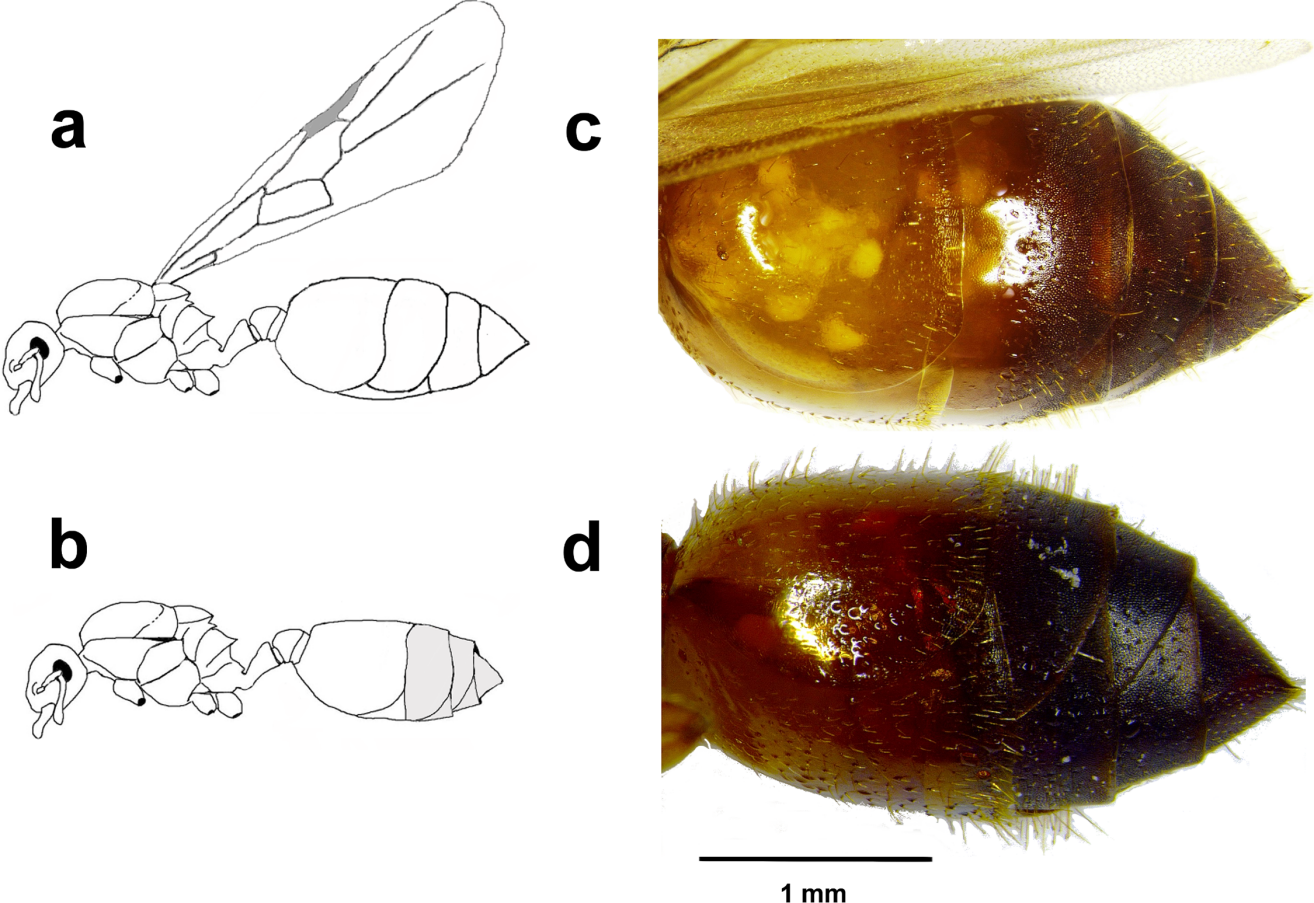
**Morphological differences between a gyne (unmated winged queen) (A,C) and a mature queen (B,D).** The gaster is yellowish in gynes while it is dark in mature queens.
